# Composite dietary antioxidant index mediates the effect of epilepsy on psychiatric disorders: results from NHANES 2013–2018

**DOI:** 10.3389/fneur.2024.1434179

**Published:** 2024-12-03

**Authors:** Xingyan He, Zhiling Li, Haotian Wu, Lifen Wang, Yuxin Zhang

**Affiliations:** ^1^Department of Pediatrics, Guangdong Provincial People's Hospital (Guangdong Academy of Medical Sciences), Southern Medical University, Guangzhou, Guangdong, China; ^2^Shantou University Medical College, Shantou, Guangdong, China; ^3^Guangdong Cardiovascular Institute, Guangdong Provincial People's Hospital, Guangdong Academy of Medical Sciences, Guangzhou, China

**Keywords:** epilepsy, psychiatric disorders, composite dietary antioxidant index, gut-brain axis, mediation effect

## Abstract

**Background:**

Psychiatric disorders is a major public health problem and epilepsy contributes significantly to depression. We aimed to explore the relationship between dietary patterns and mental illness in patients with epilepsy.

**Methods:**

The data presented here are based on the 2013–2018 National Health and Nutrition Examination Survey (NHANES). In this study, the t-test and chi-square tests or one-way analysis of variance (ANOVA) were employed for the analysis of continuous and categorical variables, respectively. Restricted cubic splines (RCSs) with four knots were employed to investigate the linear relationship and trend between the Composite Dietary Antioxidant Index (CDAI) and epilepsy and psychiatric disorders, respectively. In instances where the linear relationship was not deemed to be applicable, the CDAI was categorized into four groups based on quartiles. A logistic regression analysis was employed to investigate the relationship between epilepsy and mental disorders under four distinct models. A mediation analysis was employed to ascertain whether CDAI acted as a mediator in the relationship between epilepsy and mental disorders.

**Results:**

Patients with epilepsy had a significantly lower CDAI (*p* < 0.001) and a significantly higher prevalence of psychiatric disorders (*p* = 0.02) compared with non-epileptic patients. Mediation modeling showed that CDAI mediated between 3.17 and 5.21% of epilepsy-related psychiatric disorders. In stratified analyses, the prevalence of psychiatric disorders was increased in the second quartile subgroup and the third quartile subgroup of the CDAI dietary index in patients with epilepsy compared with non-epileptic patients.

**Conclusion:**

Our findings suggest that patients with epilepsy have a high risk of developing psychiatric disorders and that the Composite Dietary Antioxidant Index (CDAI) plays a key role in mediating the relationship between epilepsy and psychiatric disorders.

## 1 Introduction

Epilepsy is a chronic disorder in which abnormal discharges from nerve cells in the brain cause temporary brain dysfunction. In addition, a number of psychiatric disorders, including attention deficit hyperactivity disorder, depression and anxiety, are common in patients with epilepsy, and these disorders are collectively referred to as neuropsychiatric comorbidities of epilepsy. The quality of life of people with psychiatric comorbidities is often worse than that of people with epilepsy. A growing body of research also suggests that people with epilepsy are at high risk of developing psychiatric disorders such as depression, anxiety and suicide (1–3).

The interaction between diet and mental health has attracted considerable scientific and public interest in recent years (4). As a result, several studies have highlighted the potential influence of dietary factors on psychiatric disorders. Evidence suggests that a pro-inflammatory diet, characterized by high dietary inflammation index scores, may increase the risk of moderate to severe depression in patients with epilepsy (5). This finding highlights the potential for dietary factors to make an important contribution to the prevention of epilepsy complicated by psychiatric disorders.

In addition to the Dietary Inflammatory Index, the Composite Dietary Antioxidant Index (CDAI) has received increased attention in recent years. The antioxidant qualities of vitamins A, C and E, as well as the minerals selenium and zinc, are then reflected in this index (6). The initial studies on CDAI indicated a decreased probability of cancer in male smokers (7). A reduction in the risk of several chronic obstructive pulmonary diseases (COPD) has been shown to be associated with increased levels of CDAI. In addition, study has shown that post-stroke depression and all-cause mortality are independently and integrally related to dietary antioxidant intake (8, 9).

There are many mechanisms related to epilepsy complicating psychiatric disorders, and the hottest research in recent years has been on the gut-brain axis, particularly through inflammatory factor-mediated pathogenesis of psychiatric disorders. CDAI has been found to be associated with some specific inflammatory biomarkers, such as L-1β and TNFα (10). Although it has been shown that dietary inflammation index, which is associated with inflammation, plays a moderating role in epilepsy complicated by moderate to severe depression (MSD) (5). However, there are few studies on the relationship between general psychiatric disorders and epilepsy. Therefore, in this representative US sample study, we examined potential associations between dietary antioxidant levels and epilepsy and psychiatric disorders using 6,926 participants aged ≥18 years from the 2013–2018 National Health and Nutrition Examination Survey (NHANES) database.

## 2 Material

The National Health and Nutrition Examination Survey (NHANES) is a cross-sectional survey conducted by the Centers for Disease Control and prevention to document the nutritional and health status of the American population. The project surveys approximately 5,000 individuals annually, including five key domains: demographics, diet, physical examination, laboratory testing, and questionnaire-based assessments. The project is conducted in strict accordance with the principles set forth in the Declaration of Helsinki, thereby ensuring ethical conduct in research. Prior to their inclusion in the survey, all participants are required to provide written informed consent.

The NHANES protocol was approved by the National Center for Health Statistics (NCHS) Research Ethics Review Board, and all participants provided informed consent. The NHANES website^1^ provides detailed survey operation manuals, consent documents, and brochures from each survey period, along with NHANES survey data, which are accessible to the public.

## 3 Methods

### 3.1 Study participants

**Figure 1:**
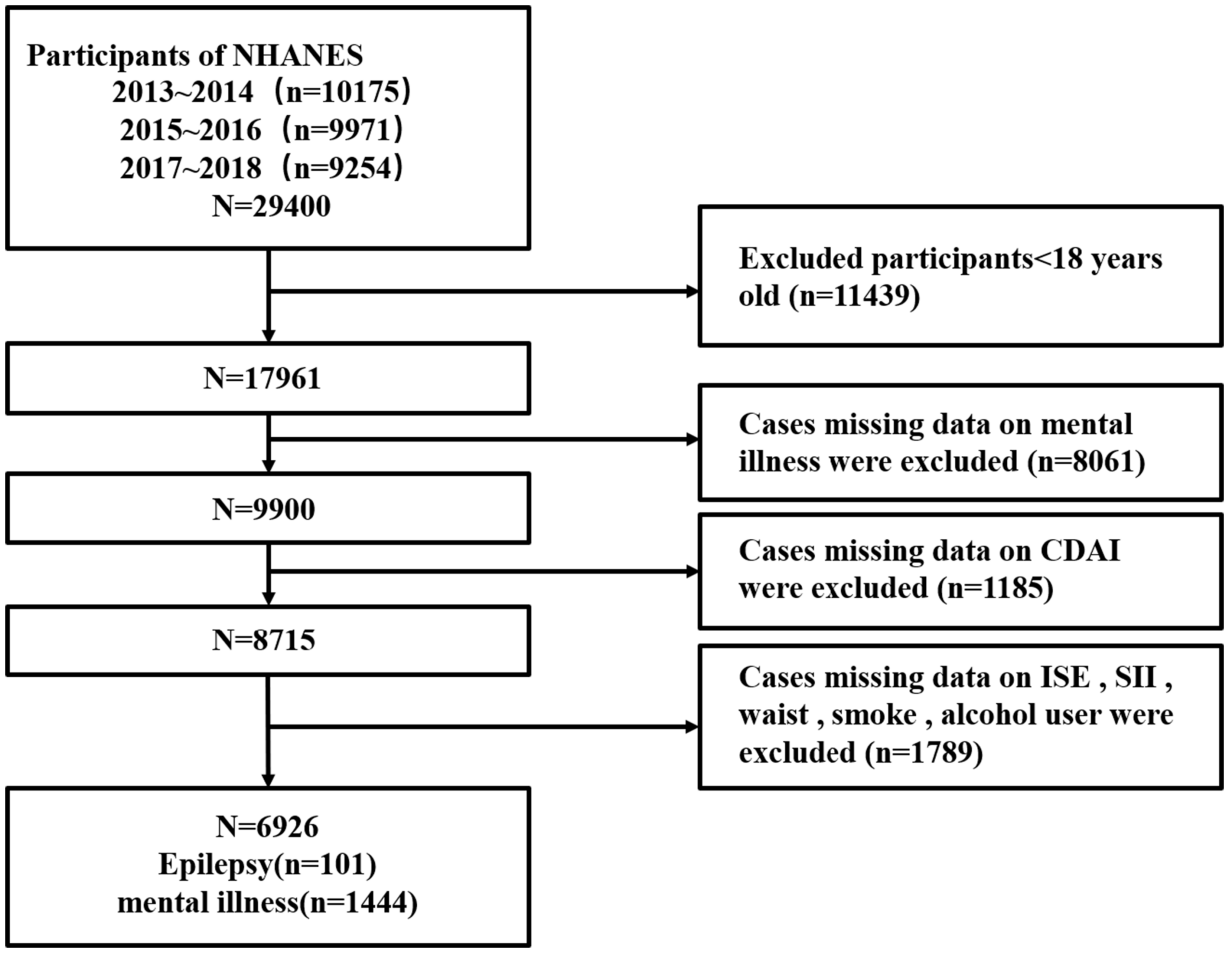
Flow chart of the selection process of the sample from NHANES 2013–2018.

The present study is based on data from the National Health and Nutrition Examination Survey (NHANES) program, which included information on epilepsy, psychiatric disorders and diet. The data were drawn from three cycles of the NHANES survey (2013–2014, 2015–2016 and 2017–2018). In the preliminary data presented, a total of 29,400 individuals participated in the survey during these cycles, with 11,439 minors aged excluded from the subsequent analysis. Among the 17,961 adults, 8,061 were excluded from the analysis due to the absence of data on psychiatric disorders. Furthermore, 2,974 participants were excluded due to the absence of data pertaining to dietary habits, demographic characteristics, and anthropometric measurements. Of the 6,926 subjects with complete data, 101 were officially identified as having epilepsy, and 1,444 were identified as having psychiatric disorders (Figure 1).

### 3.2 Assessment of epilepsy and psychiatric disorders

Epilepsy and psychiatric disorder data were obtained through face-to-face interviews conducted by investigators with participants. The disorders included in this category are schizophrenia, bipolar disorder, emotional disorders, anxiety disorders, panic disorder, obsessive-compulsive disorder, attention deficit hyperactivity disorder, conduct disorder, moderate to severe depression, and other unspecified psychiatric disorders. Participants were subjected to comprehensive questioning regarding all medications prescribed by healthcare providers within the preceding 30 days, accompanied by detailed justifications for their usage, which were frequently disease-related. With the exception of instances of moderate to severe depression, participants were classified in accordance with the International Classification of Diseases codes (F20, F29, F31.9, F32.9, F33.9, F39, F41.9, F42, F90, F91, F99), with individuals receiving medication identified as patients with either epilepsy or psychiatric disorders. Depressive symptoms were assessed using the nine-item Patient Health Questionnaire-9 (PHQ-9), comprising questions exploring the clinical manifestations of depression. This tool, which is widely employed for the purpose of depression screening, generates scores that range from 0 to 27, with responses graded on a 5-point scale. It is noteworthy that the PHQ-9 demonstrated an 88% sensitivity and specificity for the diagnosis of major depression (11). Subsequently, participants who scored above 14 were classified as experiencing moderate to severe depression.

### 3.3 Assessment of the CDAI score

The NHANES employed a non-consecutive two-day 24-h dietary recall interview to collate data on participants’ dietary intake. The initial 24-h period was recorded in person at a mobile test center, while the subsequent 24 h were reported via telephone after a period of 3–10 days. Nutrient intake was evaluated using the American Diet Research Food and Nutrition Database. In order to ascertain a representative daily dietary intake measure, the average of the 24-h intake between the two reporting periods was calculated.

The CDAI was calculated as the sum of the daily average intakes of zinc, selenium, carotenoids, vitamin A, vitamin C, and vitamin E. Standardization of each antioxidant (Xi) was conducted by subtracting the gender-specific mean (Mi) and dividing by the gender-specific standard deviation (Si) (12). Please refer to the following formulas for more details. The dietary data were analyzed using NutriDan software, as previously described in detail elsewhere (12, 13).

$$\mathrm{CDAI}=\overset{6}{\underset{i=1}{\sum }}\frac{Xi-\mu i}{si}$$

### 3.4 Assessments of covariates

We incorporated potential factors linked to epilepsy and psychiatric disorders as covariates, encompassing participants’ demographics such as age, sex, race, along with physiological metrics like body mass index (BMI) and waist circumference. Additionally, lifestyle variables including drinking status and smoking status were considered. Drinking status was delineated into categories: abstaining or mild drinking, former drinking, mild to moderate drinking (defined as ≤2 drinks/day for females or ≤3 drinks/day for males, or binge drinking 2–4 days/month), and heavy drinking (defined as ≥3 drinks/day for females or ≥4 drinks/day for males, for binge drinking ≥5 days/month). Smoking status was classified as never smoking (smoked <100 cigarettes in a lifetime), former smoking (previously smoked >100 cigarettes but currently not smoking), and current smoking (having smoked >100 cigarettes and currently smoking occasionally or daily). Moreover, the systemic immune inflammatory index (SII) was computed utilizing a formula as detailed in previous scholarly works: SII = platelet count × neutrophil count/lymphocyte count (14).

### 3.5 Statistical analysis

**Figure 2:**
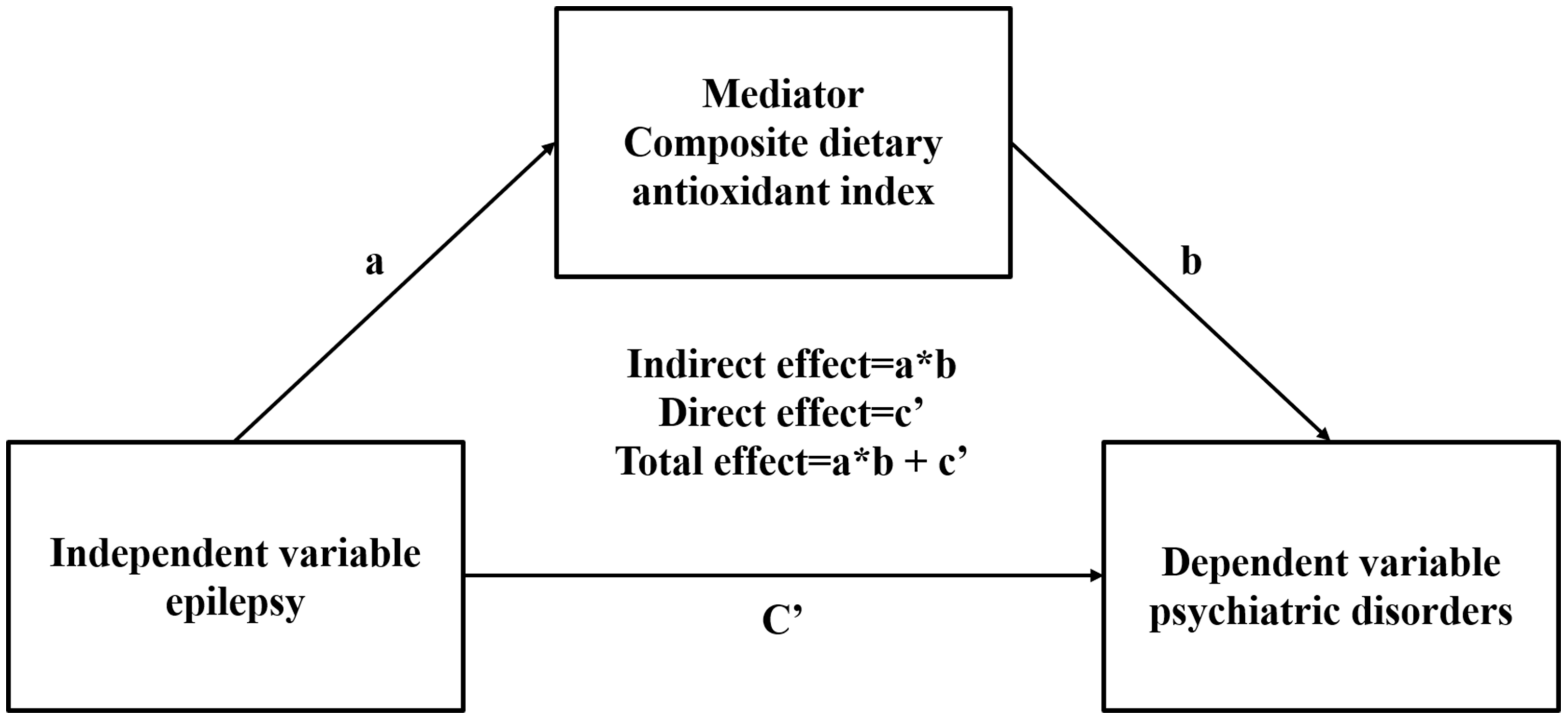
Mediation effect of composite dietary antioxidant index epilepsy and psychiatric disorders.

In order to ensure the representativeness of the entire U.S. population, weights were applied to the data collected from the previous 24 h and two additional 24-h periods. To ascertain whether there were any significant differences between the various groups, including those with and without epilepsy, as well as participants with and without psychiatric disorders, t-tests were employed for continuous variables and chi-square tests for categorical variables. Four models were constructed to adjust for potential confounding factors in the multivariate regression analysis. The models were as follows: Model 1 was the unadjusted model; Model 2 was adjusted for age, sex, and race; Model 3 was adjusted for variables from Model 2 in addition to BMI, systemic immune-inflammatory index (SII), and waist circumference; and Model 4 was adjusted for variables from Model 3 as well as smoking status and alcohol drinking status. In Model 4, restricted cubic splines (RCSs) with four knots were employed to examine the linear relationship and trend between epilepsy, psychiatric disorders, and CDAI. In models where a linear relationship was not observed, CDAI was categorized into quartiles. A multivariate logistic regression model was applied to analyze the relationship between psychiatric disorders, epilepsy, and CDAI, with epilepsy serving as the independent variable and psychiatric disorders as the dependent variable. The initial assumption was that both direct and indirect effects (mediating effects) between epilepsy and mental illness would be observed. Subsequently, CDAI was introduced as a mediating variable in the mediation model (see Figure 2). In instances where the Composite Dietary Antioxidant Index (CDAI) is associated with both epilepsy and MSD, it is presumed that there is a mediating effect of epilepsy and depression. The ratio of the indirect effect to the total effect was used to quantify the magnitude of the mediating effect. All statistical analyses were conducted using R version 4.3.2 (R Foundation for Statistical Computing). A *p*-value of less than 0.05 was deemed statistically significant.

## 4 Results

### 4.1 Sociodemographic characteristics

The demographics and health status of the participants are presented in Table 1. In comparison to the non-epilepsy group, the epilepsy group exhibited a lower mean age, a higher proportion of females, an increased likelihood of smoking, a higher prevalence of psychiatric disorders, and lower CDAI scores (*p* < 0.05, Supplementary Table 1). Substantial disparities in all examined indicators were identified between the two groups of patients categorized by psychiatric disorders (*p* < 0.05, Supplementary Table 2).

**Table 1:** Demographic and health status of participants (*n* = 6,926).

Variable	Mean (95%) or *N* (%)
Age, years	52.90 (52.17, 53.63)
Sex, *n* (%)	
Female	3,904 (57.08)
Male	3,022 (42.92)
Race, *n* (%)	
Black	1,422 (9.47)
White	3,131 (73.27)
Other	2,373 (17.26)
BMI, kg/m^2^	30.05 (29.74, 30.36)
Waist, cm	102.62 (101.92, 103.32)
SII score	535.76 (523.39, 548.13)
Alcohol, *n* (%)	
Former	1,041 (11.97)
Heavy	1,106 (17.26)
Moderate	1,125 (19.05)
No	3,654 (51.71)
Smoke, *n* (%)	
Former	1,934 (28.31)
Never	3,800 (55.82)
Now	1,192 (15.88)
CDAI score	0.69 (0.54, 0.84)
ISE, *n* (%)	
No	5,781 (80.85)
Yes	1,145 (19.15)
Epilepsy, *n* (%)	
No	6,825 (98.70)
Yes	101 (1.30)
Psychiatric disorders, *n* (%)	
No	5,482 (77.46)
Yes	1,444 (22.54)

Continuous variables are presented as weighted means (weighted standard errors) and categorical variables as raw frequencies (weighted percentages).

### 4.2 Curvilinear relation analysis

**Figure 3:**
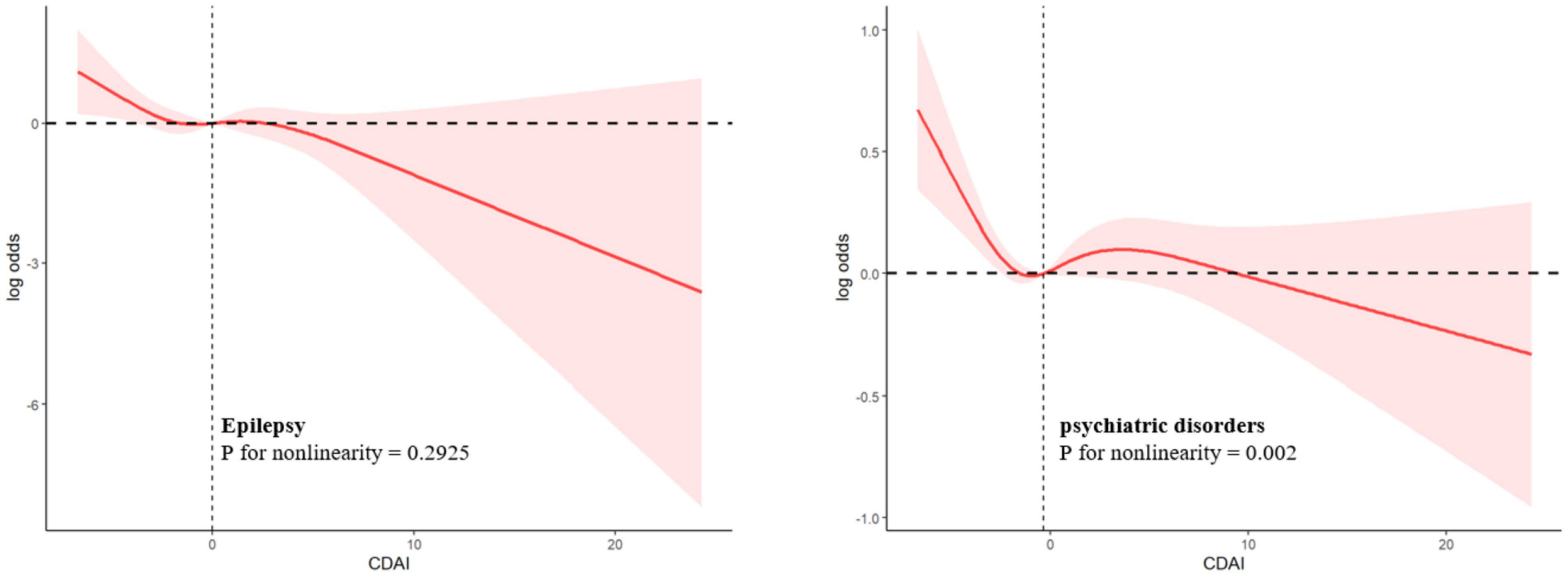
Log-transformed adjusted ORs and 95 % CIs (Model 4) of psychiatric disorders associated with the CDAI.

RCS showed that epilepsy was linearly associated with CDAI (*p* for nonlinearity = 0.2925) (Figure 3) and other dietary antioxidant diets, and with carotenoid dietary amounts in an S-shaped curve (*p* for nonlinearity = 0.0476). The relationship between psychiatric disorders and CDAI (*p* for nonlinearity = 0.002) was also S-shaped curve (Figure 3). In addition, the same characteristics can be used to describe the relationship between psychiatric disorders and dietary antioxidant dietary intake.

### 4.3 Relationship between epilepsy and composite dietary antioxidant index

Models 1, 2, 3, and 4 all showed that patients in the epilepsy group had lower levels of dietary antioxidant index, and this difference was significant (*p* < 0.01, Model 4: OR = 0.33, 95% CI, 0.15–0.73) (Table 2).

**Table 2:** Binary logistic regression analysis of the relationship between epilepsy and CDAI.

Models	Model 1	Model 2	Model 3	Model 4
Epilepsy OR (95% CI)	0.31 (0.14, 0.69)**	0.28 (0.12, 0.62)**	0.28 (0.13, 0.64)**	0.33 (0.15, 0.73)**

Model 1: unadjusted Model; Model 2: adjusted for age, gender, and race; Model 3: adjusted for variables of Model 2 as well as BMI, waist, SII, and education level; and Model 4: adjusted for variables of Model 3 as well as ISE, smoke, alcohol user.

**p < 0.01.

### 4.4 Relationship between psychiatric disorders and composite dietary antioxidant index

Taking Quartile 1 of the CDAI score as a reference, in the model 1, 2 and 3, the proportion of patients in Quartile 2 and Quartile 4 in the psychiatric disorders group were significantly lower than that in the non- psychiatric disorders group (*p* < 0.001, Q4 in Model 3: OR = 0.71, 95% CI, 0.60–0.84). This relationship is also reflected in the quartile 4 in model 4 and quartile 3 in model 1, 2 and 3, but the significance is slightly lower than that of the former (*p* < 0.05, OR = 0.80, 95%CI, 0.68–0.95). In addition, there was no significant difference between the two groups in the quartile 3 of model 4 (*p* = 0.13) (Table 3).

**Table 3:** Binary logistic regression analysis of the relationship between meantal illness and CDAI.

Models	Model 1	Model 2	Model 3	Model 4
CDAI Q OR (95%CI)				
Q1	ref	ref	ref	ref
Q2	0.74 (0.63, 0.87)***	0.71 (0.61, 0.84)***	0.71 (0.60, 0.84)***	0.75 (0.63, 0.88)***
Q3	0.83 (0.71, 0.98)*	0.80 (0.68, 0.94)*	0.80 (0.68, 0.94)*	0.88 (0.74, 1.04)
Q4	0.75 (0.64, 0.88)***	0.70 (0.60, 0.83)***	0.71 (0.60, 0.84)***	0.80 (0.68, 0.95)*

Model 1: unadjusted Model; Model 2: adjusted for age, gender, and race; Model 3: adjusted for variables of Model 2 as well as BMI, waist, SII, and education level; and Model 4: adjusted for variables of Model 3 as well as ISE, smoke, alcohol user.

*p < 0.05.

**p < 0.01.

***p < 0.001.

### 4.5 Mediation analyses

We analyzed the mediating effect of the CDAI on the relationship between epilepsy and psychiatric disorders through the mediation model. The results showed that there was a mediating effect of CDAI in all models, and all of them were statistically significant (*p* < 0.05).

In Model 4, the mediating effect mediated by the CDAI score accounted for 3.17% of the total association between epilepsy and psychiatric disorders (indirect effect = 0.019) (Table 4).

**Table 4:** Mediation effect of CDAI between epilepsy and psychiatric disorder.

Models	Direct effect	Indirect effect	Total effect	Proportion mediated (%)
Model 1	0.730***	0.030**	0.757***	3.96**
Model 2	0.712***	0.039**	0.748***	5.21**
Model 3	0.690**	0.035**	0.723***	4.84**
Model 4	0.583**	0.019*	0.600**	3.17*

Model 1: unadjusted Model; Model 2: adjusted for age, gender, and race; Model 3: adjusted for variables of Model 2 as well as BMI, waist, SII, and education level; and Model 4: adjusted for variables of Model 3 as well as ISE, smoke, alcohol user.

*p < 0.05.

**p < 0.01.

***p < 0.001.

### 4.6 Stratified analyses and interaction analyses

In stratified and interactive analysis, the CDAI were divided into four levels according to the quartile, and the results were corrected using a fully corrected model (Model 4). In stratified analysis, there was no statistically significant difference in CDAI between the Quartile1 and Quartile4 percentiles (*p* > 0.05). In Quartile 2 (*p* < 0.05, OR = 2.71, 95% CI, 1.22–6.02) and Quartile 3 (*p* < 0.05, OR = 3.49, 95% CI, 1.47–8.29), epilepsy was significantly associated with psychiatric disorders. Interaction analysis there is no interaction between epilepsy and the psychiatric disorders. Subgroup analyses that included other covariates are shown in Forest Figure (Table 5), where the interaction was found only in the drinking population.

**Table 5:** Forest plots for subgroup analysis.

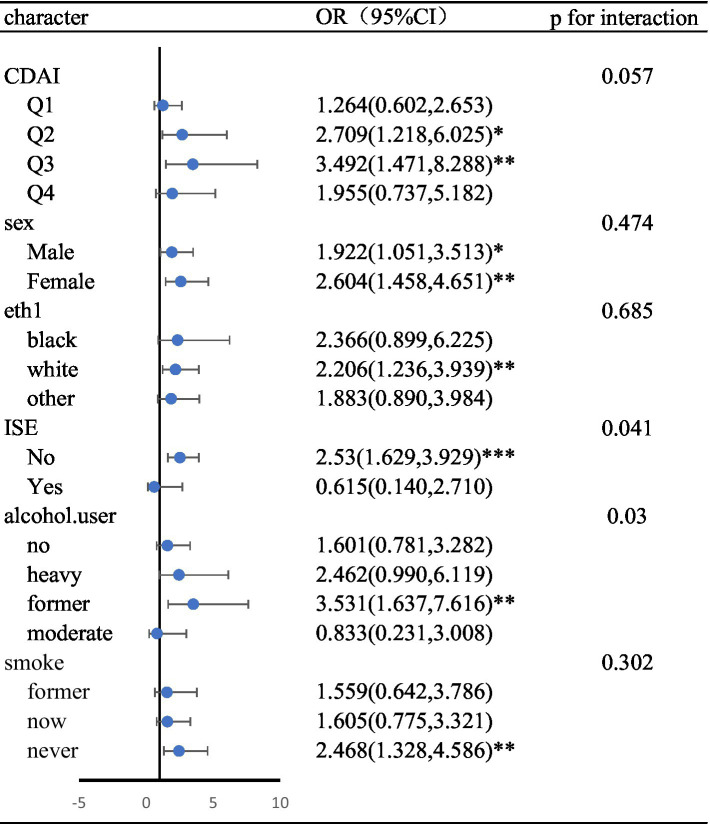

Subgroup analysis was stratified by sex, race, body mass index, drinking status, smoking status. BMI, body mass index. OR, odds ratio; 95% CI, 95% confidence interval.

*p < 0.05.

**p < 0.01.

***p < 0.001.

## 5 Discussion

The objective of this cross-sectional study was to investigate the relationship between the Composite Dietary Antioxidant Index (CDAI) and psychiatric disorders among adults aged 18 years and above who have been diagnosed with epilepsy in the United States. The data from the National Health and Nutrition Examination Survey (NHANES) for the period 2013 to 2018 was employed, with a weighted prevalence of epilepsy determined to be 1.3%. The findings of this study indicate that adults with epilepsy in the United States tend to have diets that are characterized by lower levels of dietary antioxidants. Similarly, individuals with psychiatric disorders also demonstrate a reduced intake of dietary antioxidants. The use of mediation modeling has enabled us to further elucidate the relationship between reduced levels of dietary antioxidants and concurrent psychiatric disorders in epilepsy patients, albeit with a more subtle impact.

It is not possible to ascertain whether the observed correlation between antioxidant indices and the risk of comorbid psychiatric disorders in patients with epilepsy is attributable to their intrinsic antioxidant capacity. This is due to the fact that selenium, as represented by the CDAI index, plays a role in the process of apoptosis and the initiation of DNA repair (15). Additionally, carotenoids have been demonstrated to have an important role in the processes of cell growth and development (16). The alpha and gamma tocopherols present in vitamin E have been observed to exert anti-reproductive and anti-inflammatory effects (17), respectively, which appear to be independent of their antioxidant properties. Furthermore, this index may be associated with additional known or unknown effects in food. The objective of this study was to investigate the correlation between CDAI and epilepsy and psychiatric disorders, which extend beyond antioxidant properties. Moreover Subgroup analyses are not conducted due to the potential for false-negative and false-positive results if analyses are performed in subgroups alone, given the lower prevalence of certain psychiatric disorders in the National Health and Nutrition Examination Survey (NHANES). The findings of this study indicate that a reduction in dietary antioxidant intake may be a contributing factor in the development of epilepsy-related psychiatric disorders. To gain a deeper understanding of the specific role of dietary antioxidants in these disorders, future cohort studies with larger sample sizes are necessary.

The existing literature on the lifestyles of people with epilepsy is relatively sparse, which underscores the importance of paying closer attention to the potential impact of diet and other lifestyle factors on epilepsy development, treatment, and quality of life. Earlier studies have already observed that individuals with epilepsy demonstrate a comparatively lower consumption of cheese, fruits, legumes, nuts, vitamins, and potassium, and a relatively higher consumption of sugary sodas, fats, and sodium, in comparison to healthy populations (18). Furthermore, previous literature has indicated that some adolescents with epilepsy may be at an elevated risk for developing eating disorders. Such eating disorders are frequently typified by unhealthy dietary habits and may be accompanied by dissatisfaction with one’s appearance (19). It is noteworthy that previous studies have tended to focus on specific foods or nutrients without standardizing the patient’s overall diet. It is therefore evident that a more comprehensive research approach is required in order to gain insight into the combined effects of lifestyle on health and quality of life in patients with epilepsy.

In our study, we examined the Composite Dietary Antioxidant Index (CDAI) scores of epileptic patients for the first time, with the aim of exploring the differences in dietary antioxidant levels between epileptic and non-epileptic patients. The findings revealed that patients with epilepsy exhibited lower CDAI scores than non-epileptic patients, indicating the potential for dietary antioxidant deficiencies in the former group. While all four models demonstrated a consistent reduction in CDAI scores among patients with epilepsy, the trends in the odds ratios exhibited variability following the adjustment for confounding variables (Model 4). This discrepancy may be attributed to a reduction in confounding variables in Model 4 or to a greater proportion of missing data in the covariates initially included in the model. Consequently, further studies are required to validate these findings, including larger sample sizes, longitudinal follow-up and the careful selection of appropriate covariates.

Epilepsy is most commonly comorbid with psychiatric disorders (20). Amongst the psychiatric disorders that are comorbid with epilepsy, anxiety disorders and depression are the most prevalent (1, 21). In the present study, psychiatric disorders were defined as schizophrenia, bipolar disorder, mood disorders, anxiety disorders, panic disorders, obsessive-compulsive disorder, attention deficit hyperactivity disorder, conduct disorders, moderate to severe depression, and other unspecified psychiatric disorders. Furthermore, clinical studies have demonstrated that patients with comorbid anxiety and depression and epilepsy exhibit a diminished quality of life compared to individuals with epilepsy, depression, and anxiety alone (22). Individuals with disabilities are at a significantly elevated risk of suicidal ideation and depression, with a four- to five-fold increase in the likelihood of developing these conditions compared to the general population (23). A study published in The Lancet in 2013 observed that individuals with epilepsy who present with psychiatric comorbidities exhibit an elevated risk of premature mortality (23). It is therefore of particular importance to explore an intervention that can reduce the risk of psychiatric disorders in people with epilepsy, with dietary habits representing a priority area for investigation. In recent years, it has been demonstrated that an increase in a complex dietary antioxidant intake is associated with a reduction in the risk of depression (24). Additionally, a study has indicated that an elevated intake of dietary antioxidants can prevent depressive symptoms and improve the prognosis of stroke patients (9). Our findings align with those of previous studies, which have also identified a strong association between CDAI and psychiatric disorders, particularly between the second and lowest quartiles. Furthermore, mental disorders can contribute to the formation of poor lifestyle habits, which in turn result in the consumption of foods with low antioxidant capacity. It can therefore be concluded that the implementation of an enhanced dietary management plan is an effective method of preventing and improving mental health outcomes.

The prevailing view is that individuals with epilepsy are at an elevated risk of developing depression in comparison to those without epilepsy. In light of the considerable risks associated with this comorbidity, considerable attention has been paid to strategies for the prevention and management of epilepsy-related psychiatric disorders, with a particular focus on lifestyle and dietary patterns (19). Among the extensively studied mechanisms, the brain-gut axis has been identified as a key contributor to the increased susceptibility of epileptic patients to depression (25). A disruption of the gut flora, or dysbiosis, has been identified as a potential cause of epilepsy and a trigger for seizures. The interaction between diet and gut flora demonstrates the inevitability of the influence of the gut microenvironment. There is a robust correlation between inflammatory processes and oxidative stress. Furthermore, emerging evidence indicates a correlation between CDAI and inflammatory markers, including IL-1β and TNFα, among others. This suggests that alterations in inflammatory factors can both cause and result from dysregulated gut flora. It is therefore important to consider the potential influence of CDAI on the brain via the brain-gut axis pathway. Nevertheless, there is a dearth of research examining the correlation between dietary patterns and concomitant psychiatric illnesses in individuals with epilepsy. The findings of our investigation indicate that patients with epilepsy exhibit lower levels of dietary antioxidants and a higher prevalence of psychiatric disorders compared to non-epileptic individuals. Consequently, we conducted mediation modeling to investigate the mediating role of CDAI in the context of epilepsy and psychiatric disorders. The findings revealed that CDAI exerted a mediating effect ranging from 3.17 to 5.21% across different models, indicating that diminished levels of dietary antioxidants may act as a precipitating factor for psychiatric disorders in individuals with epilepsy. Stratified analyses further unveiled disparities between epileptic patients with comorbid psychiatric disorders and their non-epileptic counterparts, particularly in terms of moderate.

## 6 Limitations

The study demonstrates significant strengths through its pioneering investigation into the role of the Composite Dietary Antioxidant Index (CDAI) in epilepsy-related psychiatric disorders. This paper employs a methodology for calculating CDAI classifications that mitigates recall bias and accounts for individual differences that may exist in cross-sectional studies. However, it is essential to acknowledge the limitations of the study. Firstly, the cross-sectional nature of the study precludes rigorous control for the various factors that may influence outcomes. Secondly, the exclusion of potentially valuable patient data due to missing information may introduce a selection bias, which could impact the study’s findings. The last, protective factors such as psychotherapy and counseling interventions were not included as covariates in this study due to the database itself and were not explored as covariates in the models. These limitations must be recognized and addressed in future research efforts.

## 7 Conclusion

The findings pertaining to adults with epilepsy have significant implications for clinical practice and public health. The findings of our study suggest a tendency among individuals with epilepsy to consume foods with lower antioxidant content, which may consequently increase the risk of developing psychiatric disorders. The mediation model employed suggests that diminished levels of antioxidant dietary intake may play a role in the manifestation of epilepsy-mediated psychiatric disorders, albeit with a relatively modest mediating influence. In conclusion, it can be stated that epilepsy has the potential to exert a direct or indirect impact on psychiatric disorders. It may be the case that augmenting antioxidant intake through dietary measures could potentially mitigate the onset of comorbid psychiatric conditions in epilepsy patients. Furthermore, additional prospective studies are necessary to substantiate this association and elucidate the potential role of diet as a modulatory factor.

## Data Availability

The datasets presented in this study can be found in online repositories. The names of the repository/repositories and accession number(s) can be found at: https://www.cdc.gov/nchs/nhanes.

## Footnotes

^1^https://www.cdc.gov/nchs/nhanes
